# Incomplete joint side tear of the subscapularis tendon with a small fragment in an adolescent tennis player: a case report

**DOI:** 10.1186/1758-2555-4-24

**Published:** 2012-07-19

**Authors:** Soki Kato, Hiroki Funasaki, Iwao Kan, Mamoru Yoshida, Kentaro Kasama, Keishi Marumo

**Affiliations:** 1 Department of Orthopaedic Surgery, The Jikei University School of Medicine, 3-25-8 Nishi-shinbashi, Minato-ku, Tokyo, 105-8461, Japan

**Keywords:** Shoulder, Lesser tuberosity, Avulsion fracture, Adolescent, Tennis

## Abstract

**Case:**

In this case report, we presented the case of an adolescent tennis player with avulsion injury of the subscapularis tendon of the right shoulder.

**Patients:**

A 17-year-old right-hand-dominant male tennis player visited our hospital complaining of pain in the anterior aspect of the right shoulder. We performed X-ray and three-dimensional computed tomography (3D-CT) and magnetic resonance imaging (MRI) scans for the diagnosis.

**Results:**

Plain radiographs did not reveal the presence of lesion; however, 3D-CT and MRI scans showed a small bony fragment located between the humeral head and the glenoid of the scapula and a high-intensity area of the subscapularis tendon. He was subsequently diagnosed with incomplete joint side tear of the subscapularis tendon with a small bony fragment. Subsequently, we performed arthroscopic excision of the bony fragment and repair of the subscapularis tendon.

**Conclusions:**

This case highlighted the presence of an injury with minor trauma associated with repeated tennis strokes in a skeletally immature patient.

## Introduction

Isolated avulsion fracture of the lesser tuberosity is a rare condition and is therefore frequently overlooked at the time of injury. Most isolated avulsion fractures of the lesser tuberosity are caused by fall or extremely strenuous sport activities. We report the case of an adolescent tennis player with chronic symptoms and incomplete joint side tear of the subscapularis tendon with a small bony fragment caused by repeated tennis strokes, with minor trauma. Previous studies have reported the cases of 4 baseball players with injuries caused by repeated throwing of the ball [1-4]. However, we encountered a patient who was injured because of repeated tennis strokes; no studies have previously reported such a case. In our case, the lesion observed was considerably smaller than that of previously reported cases of repetitive strain injury; nevertheless, it caused shoulder dysfunction. The bony fragment was arthroscopically excised, and the subscapularis tendon was repaired 4 years after the injury.

## Case report

A 17-year-old right-hand-dominant male tennis player visited our hospital complaining of pain in the anterior aspect of the right shoulder. Four years before his visit, he had experienced sudden acute pain in the anterior aspect of the right shoulder while executing a forehand stroke. Before the injury, the patient, who was a member of a tennis club at his junior high school and a private tennis club, allowed himself only 1 day of rest each month. Despite the pain, the subject continued to participate in competitive tennis for 4 years and experienced the pain especially during the follow-through phase. The pain increased gradually. One month before his visit to our hospital, he was examined by his family doctor, who advised him to rest his shoulder. However, the pain in his right shoulder persisted even while performing daily activities. Hence, he was then referred to our hospital.

Physical examination revealed tenderness at the lesser tuberosity, with exacerbation of pain by horizontal adduction and/or by internal rotation during the elevation of the humerus. Clinical evaluation of the rotator cuff muscles revealed full muscle strength of the supraspinatus and external rotator muscles. The result of the lift-off test was negative for the subscapularis tendon. The range of motion of the right and left shoulders did not exhibit any differences in forward elevation and external rotation. The range of motion in internal rotation with the arm at the side, internal and external rotations with the arm abducted at 90 degrees, internal rotation with the arm at a 90 degrees flexion, and horizontal flexion were 60, 50, 100, 20, and 100 degrees, respectively. The patient showed negative results for joint laxity test. The results of joint instability tests, such as anterior apprehension and posterior jerk tests, were negative. The results of forced horizontal flexion test was positive. Moreover, the preoperative University of California, Los Angels (UCLA) score was 25.

**Figure 1:**
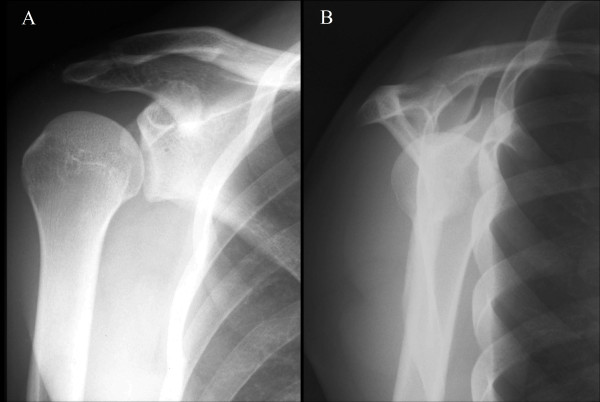
Plain radiographs of the injured shoulder showing normal appearance A, Anteroposterior and B, scapular views of the right shoulder.

**Figure 2:**
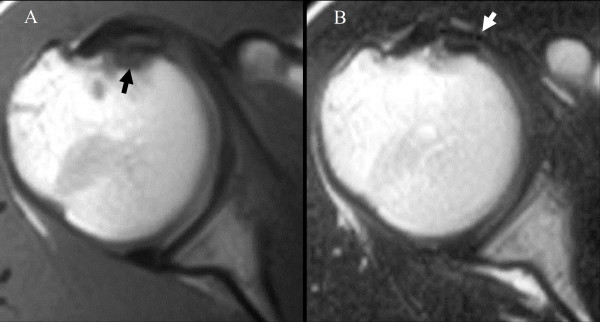
A, T1-weighted image showing a low-intensity area at the lesser tuberosity (black arrow) B, T2-weighted image showing a high-intensity area of the subscapularis tendon (white arrow).

**Figure 3:**
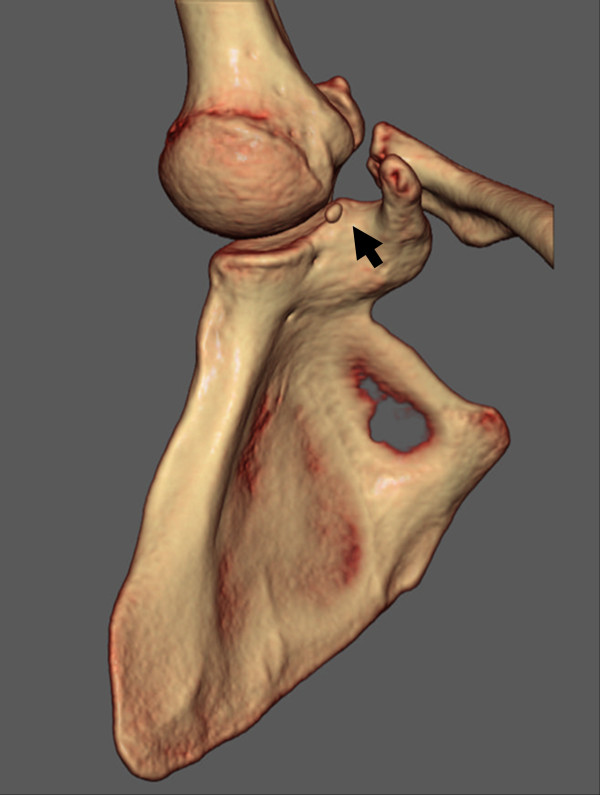
A three-dimensional-computed tomography (3D-CT) image shows a small bony fragment located between the humeral head and the glenoid of the scapula (arrow).

Plain radiographs (true anteroposterior and scapular views) of the right shoulder did not show any fracture or deformity (Figure 1). He was subsequently examined using magnetic resonance imaging (MRI). The T1-weighted images showed a low-intensity area at the lesser tuberosity, and the T2-weighted images showed a high-intensity area at the subscapularis tendon (Figure 2). Computed tomography (CT) was performed with the arm being elevated and in an internal rotation. Unfortunately, we could not confirm a small bony fragment and lesser tuberosity in the same slice because of the distinct position. However, conventional CT revealed an irregularity and osteosclerotic change at the medial edge of the lesser tuberosity. Three-dimensional CT (3D CT) revealed a small bony fragment located between the humeral head and the scapular glenoid in the position for pain provocation (Figure 3). On the basis of these results, his condition was diagnosed as an avulsion fracture of the lesser tuberosity. However, even though it was an avulsion fracture, we intended to remove the bony fragment, because it was very small and repair the tendon.

**Figure 4:**
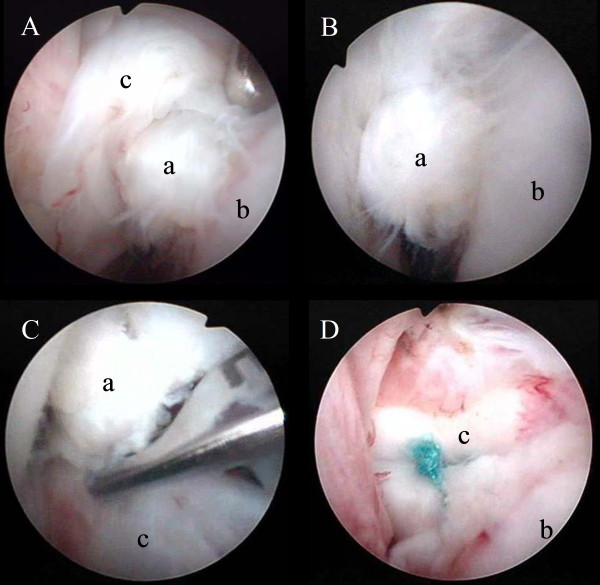
A, Arthroscopic view through the posterior portal showing articular-side tear of the subscapularis tendon (c) and a small bony fragment (a) B, When the arm was internally rotated, the small bony fragment was trapped between the humeral head (b) and the scapular glenoid C, Arthroscopic excision of the bony fragment D, Arthroscopic repair of the subscapularis tendon.

**Figure 5:**
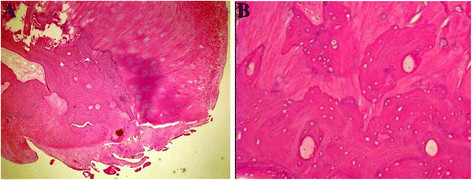
**Histological examination reveals avulsed fragment of bone surrounded by fibrocartilage.** The fragment is composed of fiber, hyaline cartilage, and cancellous bone with enucleated osteocytes (hematoxylin and eosin staining; original magnification: [**A**], 40× and [**B**], 100×).

In the operating room, the patient was maintained under general anesthesia and placed in the beach-chair position for arthroscopic removal of the fragment. Diagnostic arthroscopy was performed through a standard posterior portal. The articular side of the subscapularis tendon was torn, and an avulsion fracture was identified (Figure 4A). We could not confirm the insertion site of the deep surface of the subscapularis tendon as the fracture site, because the area was very small and covered with fibrocartilage. However, the small bony fragment was originally inserted in the deep surface of the subscapularis tendon. Subsequently, internal rotation of the arm resulted in entrapment of the small bony fragment between the humeral head and the glenoid of the scapula (Figure 4B). The bony fragment was oval and approximately 7 mm in the major axis. The long head of the biceps showed no signs of subluxation or dislocation. No further intra-articular pathological features were detected. After the anterior and anterosuperior portals were established, the small bony fragment was removed (Figure 4C). A Fastin RC suture anchor with a no. 2 Ethibond suture (Mitek, USA) was used and inserted on the side of the fracture via the anterior portal. The 2 threads protruding from the area where the anchor was inserted were introduced through the only deep surface of the subscapularis tendon by the suture relay technique. A mattress suture was then used to repair the subscapularis tendon (Figure 4D). Histological examination of the lesion showed that the avulsed bone fragment was surrounded by fibrocartilage (Figure 5).

The patient’s arm was immobilized at his side in a sling for 2 weeks after the operation. During this period, only passive flexion was permitted. After 2 weeks, active arm exercises were initiated in all planes, ensuring avoidance of provocation of pain. After a month, the patient was almost completely relieved of pain and was able to perform all the daily activities. However, preparation for college entrance examination interrupted his sport activity for a while. He entered college 2 years after the surgery and began playing tennis again at a recreational level. The postoperative range of motion was almost improved, whereas the range of motion in the horizontal flexion remained to be 100 degrees. The postoperative UCLA score also improved to 33.

## Discussion

Isolated avulsion fracture of the lesser tuberosity is an extremely rare condition and is therefore frequently overlooked in cases of injury. To the best of our knowledge, only 22 such cases in children or adolescents have been reported thus far, of which 15 patients sustained injuries during sport activities [1-15]. We encountered the case reports of 4 baseball players who sustained repetitive strain injuries caused by the throwing of the ball [1-4]. However, no study has reported cases of repetitive strain injury caused by repeated tennis strokes, as that observed in our patient.

Most isolated avulsion fractures of the lesser tuberosity are caused by falls or extremely strenuous sport activities. The mechanism of the injury is acute forced external rotation with the arm in abduction, which has been reported in children. Previous reports show large bony avulsions in which the subscapularis tendon is inserted. The subscapularis tendon was intact in all the previously reported cases in children. However, the lesions in our case resulted from low-contact sport activities, and the patient exhibited an articular side tear of the subscapularis tendon with a small bony fragment. This case is distinctly different from the previous cases. In our patient, the fragment remained trapped between the humeral head and the scapular glenoid, and the impingement was the probable cause of the shoulder pain. The patient had limited range of motion in horizontal adduction before and after the surgical treatment. The patient had posterior shoulder tightness, which might have caused altered kinematics and leading to the development of anterosuperior internal impingement during the follow-through phase [16], in addition to the avulsion injury due to contraction of the subscapularis during take-back or on hitting a ball. The patient’s immature shoulder skeletal structure was overused, leading to the injury. The patient might have been susceptible to the injury because of repeated tennis strokes.

Because the bony fragment was very small, it could not be examined by plain radiography in the true anteroposterior and scapular views. Although previous studies have reported that the axillary view is useful for the detection of smaller fragments with slight displacements [10,17,18], a plain radiograph in an axillary view was not taken at our institute. However, the CT and MRI scans were very useful for the diagnosis [10].

Patients with non-displaced avulsion fracture of the lesser tuberosity of the humerus can be treated conservatively. However, late onset of shoulder conditions, including axillary nerve palsy [6], loss of range of motion, weakness in internal rotation, and hyper-external rotation [19], caused by malunion or nonunion of the avulsed fragment after conservative treatment have been reported. Scheibel et al. [19] reported arthroscopic reduction of an isolated avulsion fracture of the lesser tuberosity. We performed arthroscopic excision of the bony fragment and repaired the subscapularis tendon and the anterior capsule. Arthroscopic surgery or open surgery could be performed in most cases. However, in our case, the lesions were located within the joint space, and the fragment was very small. Moreover, we were able to confirm the impingement by arthroscopy, which was extremely effective for the lesions located in the joint space.

In cases like our case, patients with immature skeletal structures could experience avulsion fractures of the lesser tuberosity due to minor trauma. This must be taken into consideration when such cases are encountered, and the lesion should be diagnosed and treated appropriately.

### Consent

Written informed consent was obtained from the patient for publication of this case report and any accompanying images. A copy of the written consent is available for review by the Editor-in-Chief of this journal.

## Competing interests

No potential conflict of interest declared by all authors.

## Authors’ contributions

HF, IK and SK carried out the surgical treatment and HF, MY, KK and SK contributed to the follow-up examinations in an outpatient clinic. HF, SK and KM co-wrote the paper, discussed the results and commented on the manuscript. All authors have read and approved the final manuscript.
